# Tree-Based Position Weight Matrix Approach to Model Transcription Factor Binding Site Profiles

**DOI:** 10.1371/journal.pone.0024210

**Published:** 2011-09-02

**Authors:** Yingtao Bi, Hyunsoo Kim, Ravi Gupta, Ramana V. Davuluri

**Affiliations:** Molecular and Cellular Oncogenesis Program, Center for Systems and Computational Biology, The Wistar Institute, Philadelphia, Pennsylvania, United States of America; University of Leuven, Belgium

## Abstract

Most of the position weight matrix (PWM) based bioinformatics methods developed to predict transcription factor binding sites (TFBS) assume each nucleotide in the sequence motif contributes independently to the interaction between protein and DNA sequence, usually producing high false positive predictions. The increasing availability of TF enrichment profiles from recent ChIP-Seq methodology facilitates the investigation of dependent structure and accurate prediction of TFBSs. We develop a novel Tree-based PWM (TPWM) approach to accurately model the interaction between TF and its binding site. The whole tree-structured PWM could be considered as a mixture of different conditional-PWMs. We propose a discriminative approach, called TPD (TPWM based Discriminative Approach), to construct the TPWM from the ChIP-Seq data with a pre-existing PWM. To achieve the maximum discriminative power between the positive and negative datasets, the cutoff value is determined based on the Matthew Correlation Coefficient (MCC). The resulting TPWMs are evaluated with respect to accuracy on extensive synthetic datasets. We then apply our TPWM discriminative approach on several real ChIP-Seq datasets to refine the current TFBS models stored in the TRANSFAC database. Experiments on both the simulated and real ChIP-Seq data show that the proposed method starting from existing PWM has consistently better performance than existing tools in detecting the TFBSs. The improved accuracy is the result of modelling the complete dependent structure of the motifs and better prediction of true positive rate. The findings could lead to better understanding of the mechanisms of TF-DNA interactions.

## Introduction

Transcription factors are group of proteins that participate in gene regulation by binding to specific short DNA sequences, known as transcription factor binding sites (TFBS). Accurate identification of the TFBSs is the first and perhaps the most critical step in modeling the gene regulatory mechanisms from datasets generated by recent high-throughput approaches, such as ChIP-Seq/chip [1]. TFBSs are usually short and degenerated at multiple positions. Although numerous computational approaches to predict the TFBSs have been proposed in recent years, the high false positive rate is still a problem. The problem of predicting TFBSs still remains as one of the hard problems in computational biology [2], [3], [4], [5], [6]. Depending on the representation of the TFBSs, the computational prediction methods fall into three broad classes: the PWM-based approaches [7], [8], consensus sequences-based or regular expressions-based approaches [9], [10], [11] and feature-based methods [12], [13].

Generally, PWM-based approaches assume independence between the base positions of the sequence motif and suffer from high false positive rates. However, recent studies have shown that the independent assumption is not true and modeling the dependencies in TFBSs could lead to better predictions [14]. Examples include feature-based method [13], [15], HMM-based method [16], [17], Markov Chain based method [18]. These methods could account for the strong nearest-neighbor (adjacent or local) dependencies, but still fail to incorporate potentially important longer-range interactions.

Long-range dependency in the DNA motif could be important due to the 3-D structure of the TFBS-proteins binding complex [19]. The 3-D structure of the complex makes the cooperation between non-adjacent nucleotide positions possible. Several approaches have been proposed to incorporate such dependency. Examples include Optimized Markov chain model [20], MDD [21], PVLMM [22], Bayesian Network [23], Generalized PWM [24], non-parametric method [25]. Generalized PWM extends the original PWM model to include pairs of correlated positions and uses MCMC algorithm to sample in the model space. Optimized Markov chain model reorders the nucleotides positions of the motif such that the most significantly dependent positions become pairs of adjacent positions, and then a Markov model is trained using the reordered training sequences.

In addition, the recent experimental studies have shown that a particular transcription factor may have different binding profiles under different condition, for example in the presence of different co-regulators, which suggests that the overall binding profile could be context-dependent or a mixture of different subclasses [26]. Previous studies showed that for factors which bind to divergent binding sites, mixture of multiple PWMs increase performance of the prediction [27], [28]. The potential cluster structure could make the dependent structure of the motif complicated. Thus, there is a growing need to develop methods to model the biological complexity of binding sites sequences beyond a single independent model, especially methods which could efficiently and robustly utilize the huge information provided by ChIP-Seq/chip experiments.

Although the Bayesian network modeling can capture the complicated dependent structure of the TFBS, the structure learning of the network is very complex and time-consuming, and the predicted network is very unlikely to be the true model [29]. Both MDD and PVLMM can also capture the complicated dependent structure. MDD iteratively splits the training data into a binary tree and different conditional independent models are fit to the leaf nodes of the tree. PVLMM extends the optimized Markov model by introducing variable length Markov models, which allow for different order of dependency in the reordered motif. However, all the existing methods are developed on a set of aligned exact known TFBSs or splice sites. None of these methods are optimized for analysis of large set of TF binding profiles derived in ChIP-seq experiments.

ChIP-Seq experimental methodology combines chromatin immunoprecipitation (ChIP) of a protein with massive parallel sequencing of the retrieved genomic sequences, which are mapped back to the reference genome to obtain significant peaks [1], [30]. The sequences within those peaks are expected to be enriched with TFBS of the corresponding TF of interest. ChIP-Seq is a genome scale experiment, which provides a comprehensive analysis of protein-DNA interactions. The sequence enrichment profiles provide excellent opportunity to model the dependent structure of the TFBS motif. However, with ChIP-seq technology, TF bound genomic regions cannot be identified solely on the presence of sequence enrichment on a genomic location, due to the non-specificity of the antibody, indirect binding of TF through protein-protein interactions, sequencing error, etc. Further computational analyses are needed to extract precise TFBS location. Most of existing computational approaches are not designed for processing huge data sets. Applying them on all the sequences are very time consuming. In practice, usually just the top candidate peaks are submitted to those algorithms, rather than using all of the sequence data from significant peaks [31].

Recently, several groups have started to develop prediction tools utilizing the huge information provided by ChIP-Seq. HMS extends the generalized PWM method to incorporate peak height information to aid motif identification. In order to handle a large number of input sequences and increase computational speed, it uses a novel Gibbs sampling method, where the motif alignment variables are sampled from a small proportion of top sequences, rather than from all sequences [32]. ChIPMunk is an iterative algorithm which can take into account the peak shape from ChIP-Seq data and extract the single optimal motif from large data sets like ChIP-Seq [33]. Gapped PWM examines the flexibility to allow variable length motif models utilizing the ChIP-Seq data [34]. However none of these methods can model the long range dependency between different positions in the binding sites. The huge amount information generated by ChIP-Seq experiments gives an excellent opportunity to refine those PWMs stored in transcription factor databases, like TRANSFAC [35] or JASPAR [36]. Usually the stored PWMs come from a limited number of experimentally verified TFBSs and do not truly reflect the general binding affinity of transcription factors. A recent study refined those stored PWMs based on a discriminative approach, however they assume independent motif models and do not utilize the vast information provided by ChIP-Seq experiments [37]. To this end, we developed a novel Tree-based PWM approach to accurately model the binding profile and also proposed a discriminative approach (TPD) to construct it from the ChIP-Seq data.

The paper is organized as follows. In section 2, we describe how to construct the TPWM and then the detailed implementation of TPD. In section 3, we evaluate the performance of TPD on both simulated and real biological data.

## Methods

### Modelling motif dependent structure by TPWM

Here we describe a Tree-based PWM approach to model the dependent structure of a motif. This approach is inspired by the maximal dependence decomposition (MDD) method discussed in [21], which seeks to account for the most significant non-adjacent as well as adjacent dependencies in the pre-mRNA splicing signals, using an iterative subdivision of the sequence data. TPWM is inspired by MDD but has been augmented by a number of critical modifications that make it suitable for modelling TFBS.

MDD can model long-range interaction and capture the most significant dependencies between positions, provided sufficient data are available to do so reliably. MDD assumes the consensus sequence of a motif, which defines exactly what sequences of letters constitute a match, is known and a Bernoulli random variable at each position of the motif to model whether the sequence letter at this position is consistent with the consensus sequence or not. MDD also applies the chi-square test to measure the dependence between any two positions. However, chi-square test may fail when there are zeros in the contingency table (the asymptotic distribution of the test statistic is no longer chi-square). In the following proposed TPWM approach, we assume the consensus sequence of the true motif is unknown and a multinomial random variable at each position to model the possible nucleotides at that position. We also utilize total variation distance or Hamming distance [32], instead of chi-square, to measure the dependence.

Let 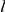
denote the motif length and 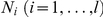 be a multinomial random variable, whose possible values are the 4 nucleotides at position 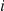 of the motif. Given a set of 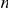 aligned TFBSs denoted by 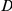, each of which has length 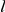, the following four steps are applied:

(i) For each pair of positions 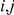 with 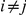, first estimates the distribution of the bivariate random variables 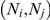 under null hypothesis (independent between position 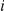 and 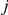): 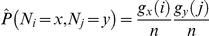 where 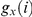
represents the number of TFBSs whose i-th position is occupied by nucleotide 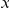. Then estimates the distribution of 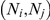 under alternative (these two positions are dependent): 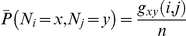, where 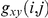 represents the number of TFBSs whose i-th and j-th positions are occupied by nucleotides 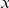 and 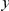, respectively.

(ii) Calculate the Hamming distance between the two kinds of estimations in (i) as 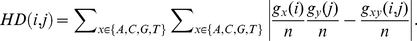 Larger 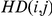 denotes stronger dependency between position 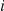 and 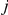.

(iii) For each position 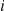 calculate the sum 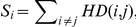 which can be considered as a measure of the amount of dependence between the variable 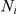 and the nucleotides at all other positions of the motif.

(iiii) Choose the value 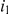
such that 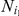 is dependent with at least one of the other positions and 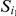
is a maximal. Position 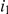 is called maximal dependent position. Then partition 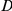 into four subsets according the nucleotide at position 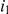: 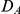, all sequences which has nucleotide A at position 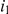; and 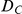, 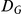 and 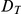 which has nucleotide C, G and T at position 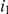 respectively. Only those subsets which have the number of sequences larger than a preset minimum value are considered as splitting branches. The preset minimum value is used to avoid unreliable estimations of the conditional probabilities after further subdivision.

Next repeat the four steps on the splitting branches and on branches thereof, and so on, yielding a tree based PWM (each non-leaf node has at most four children). The height of this tree is at most 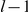 and this process of subdivision is carried out successively on each of those splitting branches until no significant dependencies between positions in a branch are detected (Here, the positions 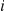
and 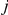 are considered to be dependent if 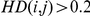 [32] ). Thus a leaf node is not split either due to no dependency detected or the number of the sequences in each of the subsets is less than the preset minimum value.

Finally, separate independent PWM models are derived for all the leaf nodes of the tree, and these are combined with the probability distributions at each non-leaf node to form a composite model. This TPWM is updated by the discriminative approach proposed in the following section.

### TPD Algorithm

Recently, an algorithm to refine a motif that best discriminates between a positive set of sequences and a background one, using ChIP-chip data was presented [37]. The proposed algorithm controls the false positive rate by fixing the percentage of binding sites predicted in the background dataset (or negative dataset). In that paper 30% was chosen by the authors based on the best compromise between the positive and the negative sets.

Here, we propose an alternative discriminative approach (TPD) to construct the TPWM from an initial input PWM using ChIP-Seq data. Instead of fixing the false positive rate, we utilize Matthew Correlation Coeffication (MCC) to determine the best separation between the positive and negative sets. The MCC is defined as follow: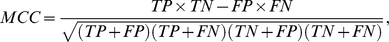 where 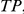 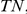 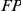 and 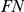 stand for true positive, true negative, false positive and false negative, respectively.

**Figure 1:**
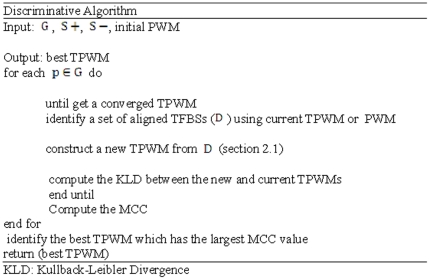
Flowchart of TPD algorithm.

The main inputs of the algorithm are two sets of sequences: 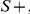 supposed to contain the sequences enriched with binding sites, and 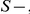
supposed to contain sequences that do not have any binding sites. In addition to 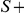
and 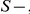 by utilizing an initial PWM and a predefined set of false positive rates 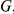 the algorithm outputs a TPWM, which best discriminates 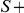 and 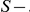 This algorithm first constructs TPWM for each false positive rate in 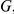 and then outputs the best TPWM that has the largest MCC value. The flowchart of the proposed algorithm is presented in Figure 1, and we assume that each sequence in 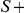 may contain at most one binding site.

For each of the inner iteration, the aligned TFBSs (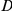) are obtained by scanning every sequence in the positive set 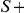 using current TPWM and the cutoff value identified by scanning 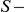 corresponding to the current false positive rate 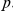 The convergence of the inner iteration is achieved if the KLD value is less than a given threshold, 0.001 in our case. The initial PWM is given by the corresponding motif patterns stored in TRANSFAC or JASPAR databases.

## Results

### Synthetic Datasets

To evaluate the ability of TPD for identifying the correct motif under different conditions, we conducted extensive simulation studies. We consider two simulation scenarios: (i) generating the nucleotides of the motif independently, (ii) assuming dependency in some positions.

#### Independent motif model Simulations

With respect to the independent model, following the simulation scheme employed in [38] , four motif models are manually created (Figure S1 and Table S1 ), representing two different motif widths (10 bp and 20 bp) and two different degrees of conservation (strong and weak) measured by 
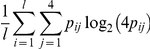
, where 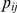 denotes the elements in the PWM matrix (if 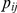
is zero, then that term is taken as zero). The logo plots are generated using R package ‘seqLogo’ [39]. Finally, two different motif abundance schemes (Table S2) were considered for a total of eight combinations in the simulation study. “Abundant scheme” means that each sequence contains the motif with probability equal to 0.8, while “weak scheme” with probability equal to 0.5. Totally, there are eight simulation settings, which cover a wide range of scenarios.

For each setting, we simulate 10 test datasets. Each dataset is generated in the following manner. A set of 3000 sequences, each of length 200 bp, are generated from a third-order Markov model with parameters estimated from the collection of 5 kb promoter sequences of UCSC known genes in the human genome [40]. Following the abundance schemes mentioned previously, we then insert the corresponding motif into the sequences of the test data set at random positions. Ten negative datasets are generated by randomly shuffling the sequences of the 10 test datasets once using Fisher-Yates shuffle algorithm.

We then run TPD on the positive and the corresponding negative datasets, using the consensus sequences of the corresponding motif model as the initial input. We select HMS, ChIPMunk (the two latest motif discovery algorithms) and the well-known motif discovery tool, MEME as benchmark algorithms for comparative analysis. To have a fair comparison, the consensus sequence is also used as prior input of MEME. The HMS is used with the -nobase option to specify a uniform prior distribution for the motif start location and with the independent setting option, dep  = 1. All the other parameters are set to be recommended values by the authors. ChIPMunk is set to be in simple mode.

**Figure 2:**
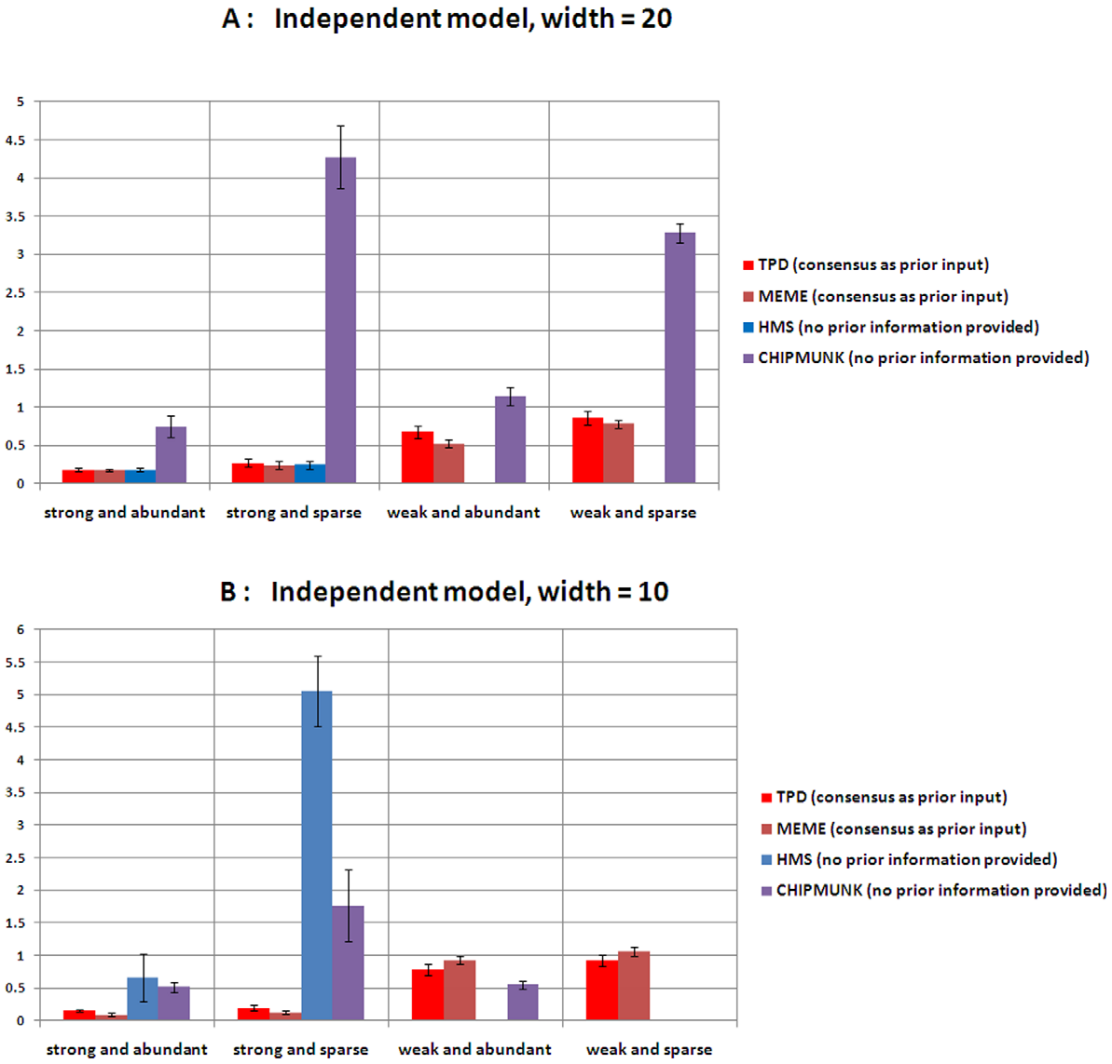
Performance comparison on simulated data with independent motif model. The y-axis represents the sum of the absolute differences between the true probabilities and their predictions from the corresponding motif finding methods. The error bar represents the standard deviation of the differences across 10 datasets (A) Independent, motif width = 20 bp. (B) Motif width = 10 bp.

We compare performance on motif pattern prediction accuracy, which is defined as the sum of the absolute differences between the true probabilities and their predictions: 
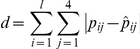
, where 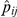 denotes the prediction of 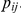 The lower the 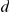
is, the better performance of the method achieves. The performances of different methods under each setting are shown in Figure 2. The missing bars under some settings indicate that the corresponding methods fail to identify the true motif. ChIPMunk performs significantly worse if the abundance level is low and performs relatively better under the weak motif model. HMS performs well for strong motif models and fails to identify the correct motif for both weak cases. TPD does not detect any dependent positions for any test dataset. Both TPD and MEME outperform the others and reliably identify the true motifs under all settings. It can be concluded that TPD and MEME are competitive under independent models.

#### Dependent motif model Simulations

With respect to the dependent motif model, totally we create six different motif patterns, representing different motif widths, different motif information content and different numbers of correlation positions (Table S3).

The dependent motif patterns are manually generated by setting some positions in the independent motif models correlated together. For the motif model with width equal to 10, two positions are set to be correlated together and the joint distributions of these two positions are specified in Table S4. For the motif model with width equal to 20, four or six positions are set to be correlated together (Table S5 and Table S6). Totally we have six different motif patterns and similar to the independent simulation study, two different motif abundance schemes are considered for a total of 12 combinations in the “dependent” simulation study.

**Figure 3:**
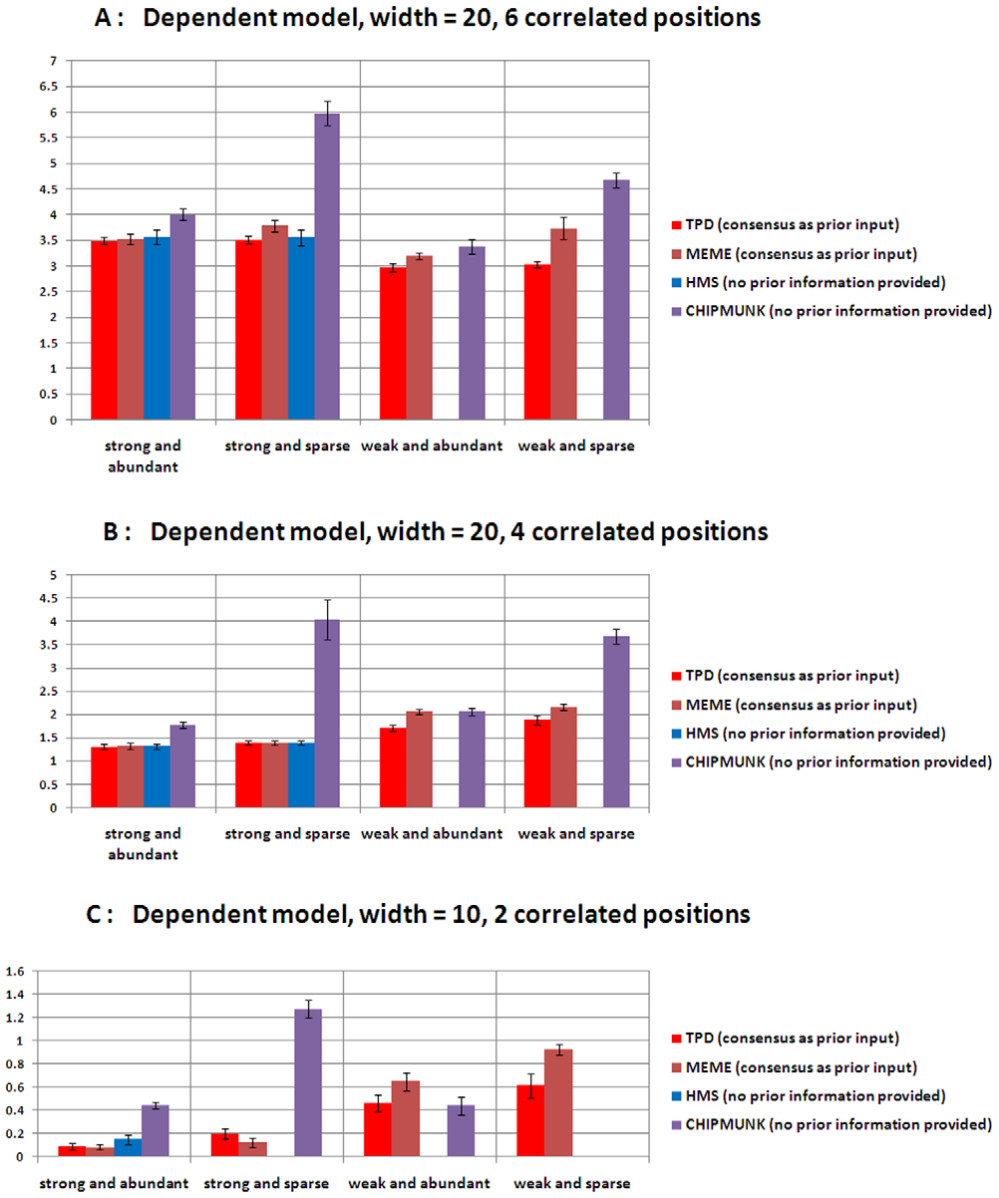
Performance comparison on simulated data with dependent motif model. The y-axis represents the sum of the absolute differences between the true probabilities and their predictions from the corresponding motif finding methods. The error bar represents the standard deviation of the differences across 10 datasets. (A) Motif width = 20 bp, 6 correlated positions (B) Motif width = 20 bp, 4 correlated positions (C) Motif width = 10 bp, 2 correlated positions.

**Figure 4:**
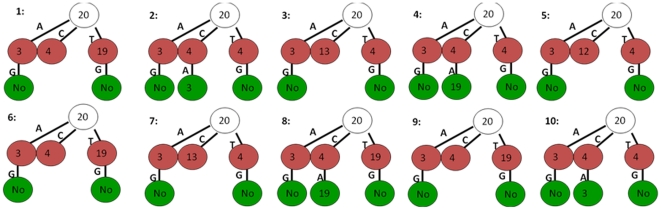
TPWMs of the predicted simulated dependent motif model by TPD for the strong, abundant and 6 correlated positions (3,4,12,13,19,20) model for each of the 10 test datasets.

In this simulation study, we run HMS with option “dep  = 3” to specify up to 3 correlated positions. Both MEME and ChIPMunk assume that all the positions are independent and are used as the same parameter setting as the independent scenario. The motif pattern prediction accuracy is defined as the sum of the absolute differences between the true marginal probabilities and their predictions. TPD outperforms all the other methods for width = 20 (Figure 3A and 3B). Both TPD and MEME successfully identify the correct motifs under all situations. For width = 10 and strong motif pattern, MEME outperforms TPD based on the accuracy of estimating marginal probabilities. The accuracy based on marginal probabilities does not reflect the predicted dependent structure. The dependent structures obtained by TPD for the setting of abundant, 20 bp width and strong motif pattern with 6 correlated positions are shown in Figure 4.

In Figure 4, the tree diagram is used to represent the dependent structure of the identified motif pattern. The number in each node of the tree denotes the identified maximal dependent position. The non-leaf nodes are split at those positions. Each non-leaf node has at most four branches, which are in the order of “A”, “C”, “G” and “T from left to right. “No” in some of the leaf nodes indicates that there is no significant dependencies detected in this node, such that no further splitting is necessary. Other leaf nodes are not split due to the number of sequences in every subset is less than the preset minimum value. For example, the first tree in Figure 4 shows that position 20 is the maximal dependent position based on all the 3000 simulated sequences. For those sequences which have nucleotide "A" at position 20, position 3 is the maximal dependent position. Similar for those sequences which have "C" or "T" at position 20, position 4 or 19 is the maximal dependent position. Due to the number of sequences which have "G" at position 20 is less than a preset minimum number, the "G" branch is missing. For the subset of sequences which have "C" at position 20, there is no further subdivision at position 4 because the number of sequences in every further subset is smaller than the preset minimum value. The subset of sequences which have "A" at position 20 is splitted at position 3 and no significant dependencies between positions in "G" branch are detected. Again the other three branches are missing because the number of sequences is smaller than preset minimum value. Figure 4 shows that only those correlated positions in the true motif model are recovered by TPD and the first split position is at 20 for all these ten predicted TPWMs. The splitting positions at the second level of the trees vary a little bit, since the random generation of the datasets. Some of the leaf nodes are not split due to the small number of sequences. We increase the total number of sequence from 3000 to 6000 for this simulation setting and run TPD again. The results are shown in Figure S2. The predicted TPWMs in Figure S2 show that more number of sequence fed to TPD could lead to the prediction of more complete dependent structure. It can be concluded that TPD can successfully detect the existing complicated dependent structure of the motif, provided sufficient data are available.

The better performance achieved by TPD is due to the modelling of the complicated dependent structure within the motif by TPWM and the accurate prediction of the true positive rate based on the maximization of the MCC value (Table S7 and Table S8).

### Real ChIP-Seq Datasets

To further evaluate our method, we tested TPD on three published ChIP-Seq datasets. The datasets were generated using antibodies against the neuron-restrictive silencer factor (NRSF) [41], CCCTF-binding factor (CTCF) [42] and Estrogen receptor alpha (ER-alpha) [32]. NRSF is an essential vertebrate zinc finger TF involved in diverse functions, including repressing neuronal genes in non-neuronal tissues, develop neurons in the brain, smooth muscles development, and play important roles in cancers or other diseases [26], [43]. CTCF is an evolutionarily conserved zinc finger TF, involved in a wide variety of functions, including negative regulation of MYC, insulator activity and repressing the insulin-like growth factor 2 gene [44]. ER-alpha is a ligand-activated TF known to play important role in breast cancer development. Thus, identifying the correct target genes of these three TFs and refining the motif patterns of them are of significant interest. The ChIP-Seq data sets are downloaded from www.sph.umich.edu/csg/qin/HMS/ and the number of candidate sequences is 22159 for CTCF, 10049 for ER-alpha and 4982 for NRSF.

**Figure 5:**
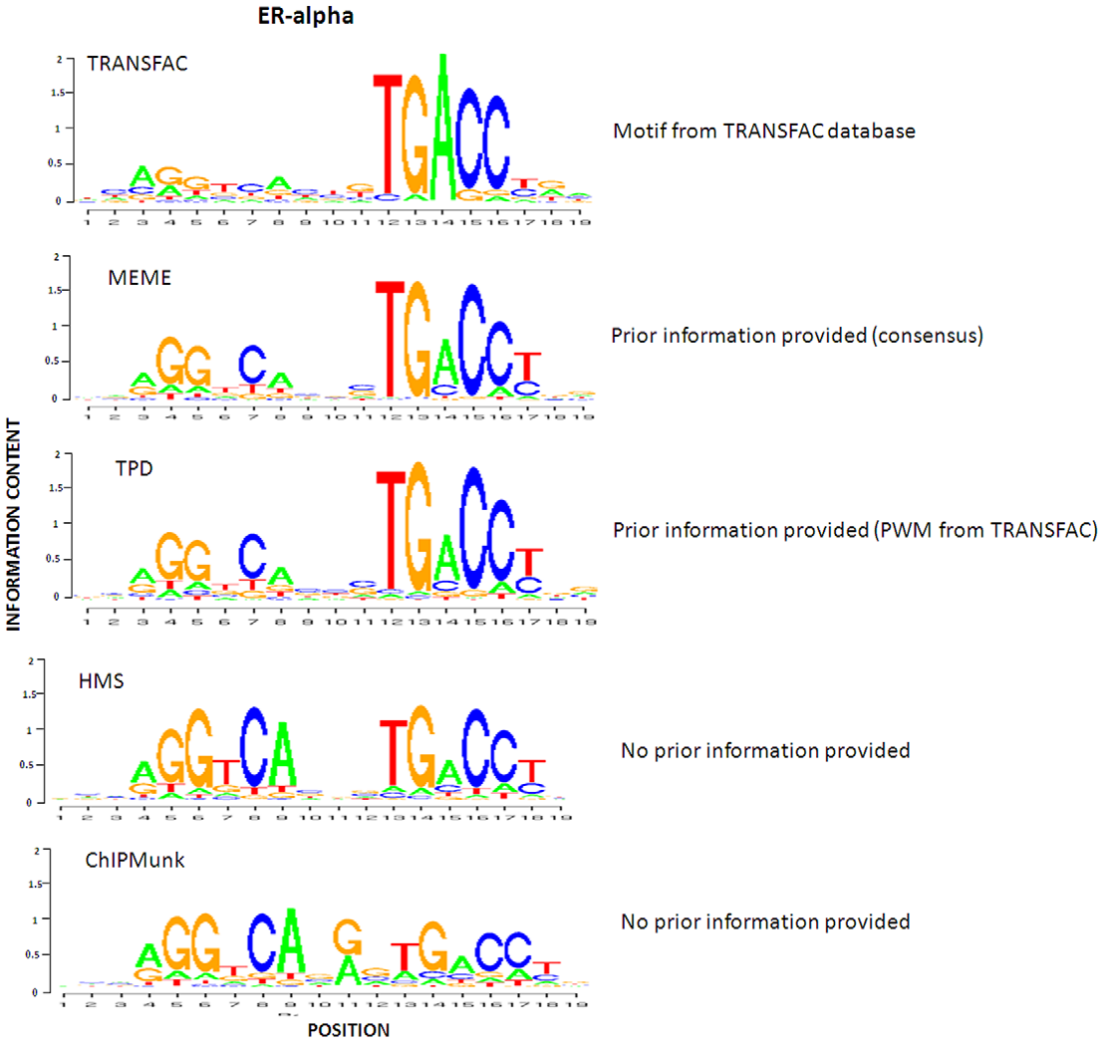
Comparison of ER-alpha motif patterns identified by TPD, MEME, HMS, and ChIPMunk, as well as known pattern stored in TRANSFAC database.

**Figure 6:**
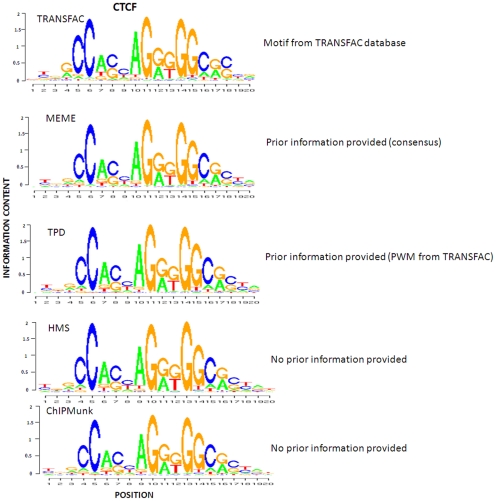
Comparison of CTCF motif patterns identified by TPD, MEME, HMS, and ChIPMunk, as well as known pattern stored in TRANSFAC database.

**Figure 7:**
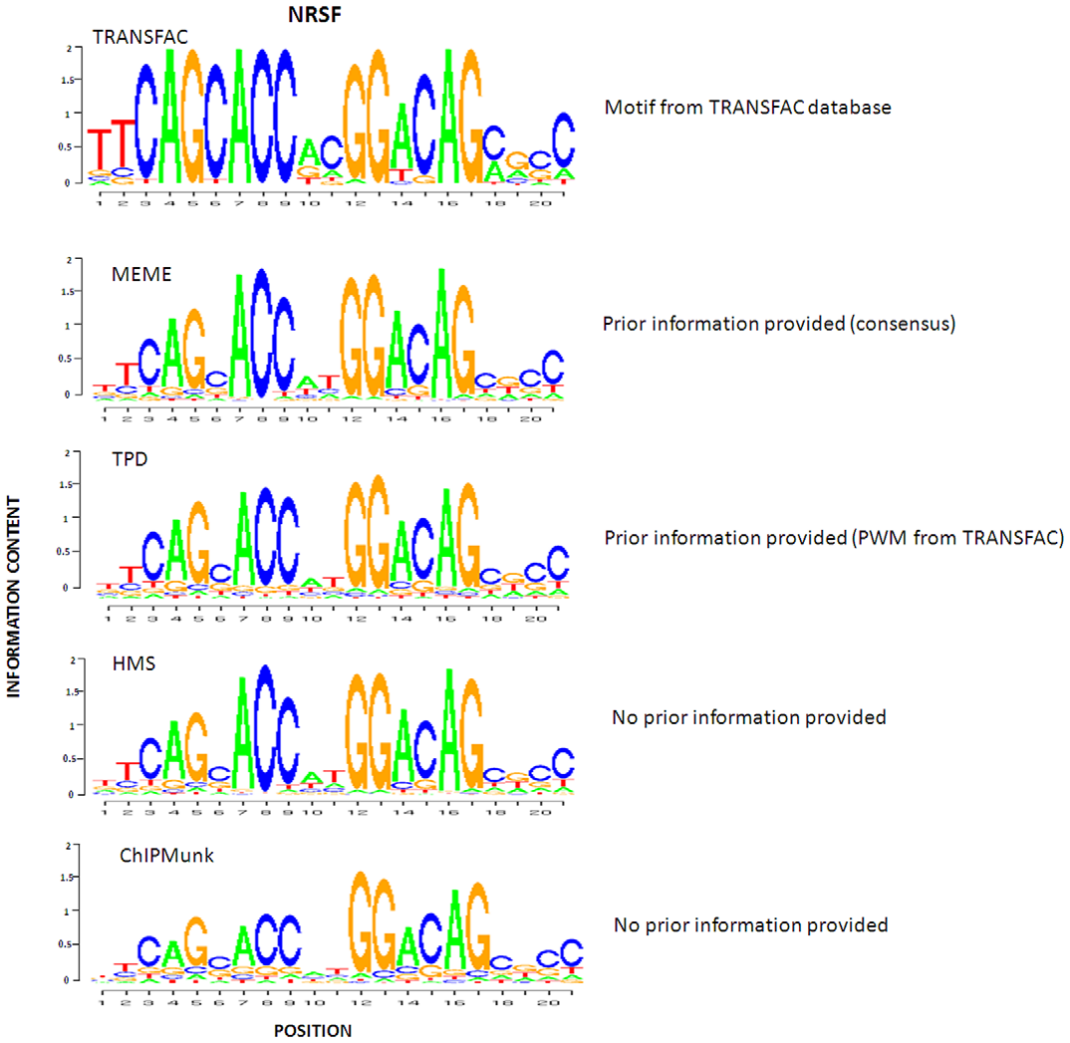
Comparison of NRSF motif patterns identified by TPD, MEME, HMS, and ChIPMunk, as well as known pattern stored in TRANSFAC database.

Similar to the simulation study, we selected HMS, ChIPMunk and MEME for comparative analysis. We also compared our method to TRANSFAC stored PWMs. We fed the entire set of sequences to all these four programs. MEME was used with –con option with the corresponding consensus sequences from TRANSFAC as prior input. HMS was used with two different versions, HMS-INDEP and HMS-DEP. TPD was used with the PWM stored in TRANSFAC database as initial input. The identified motif patterns of these three factors are presented in Figures 5, 6 and 7.

**Figure 8:**
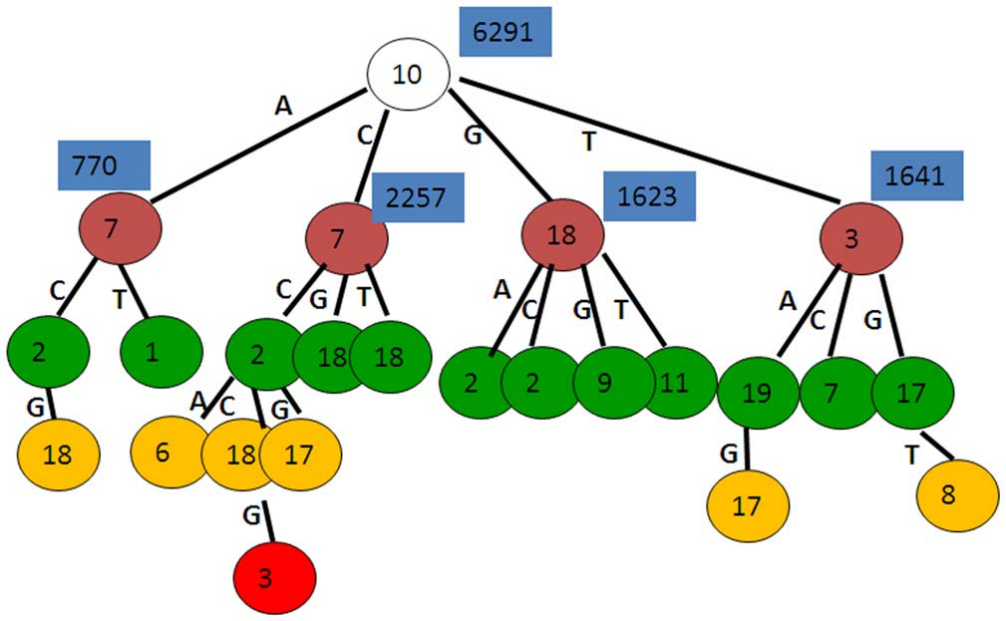
Predicted TPWM by TPD for ER-alpha.

With respect to ER-alpha factor shown in Figure 5, HMS obtained a more palindromic motif relative to TPD, but lower information content. TPD and MEME achieve quite similar motif patterns. The information content of the left half site of the motif identified by TPD and MEME is between TRANSFAC PWM and the one identified by HMS. The motif pattern identified by ChIPMunk has a gap position with high information content compared with the ones identified by other methods. With respect to CTCF shown in Figure 6, all the motif patterns are highly consistent. With respect to NRSF shown in Figure 7, the motif model identified by TPD, MEME, HMS and ChIPMunk all are less conserved relative to TRANSFAC PWM. The one predicted by ChIPMunk has the lowest information content. Note that the PWM used to generate the logo plot for either TPD or HMS is computed based on all the binding sites identified by them, respectively and can't demonstrate the dependent structure of the motifs. The dependent structure of the motif pattern (TPWM) for ER-alpha identified by TPD is shown in Figure 8.

**Figure 9:**
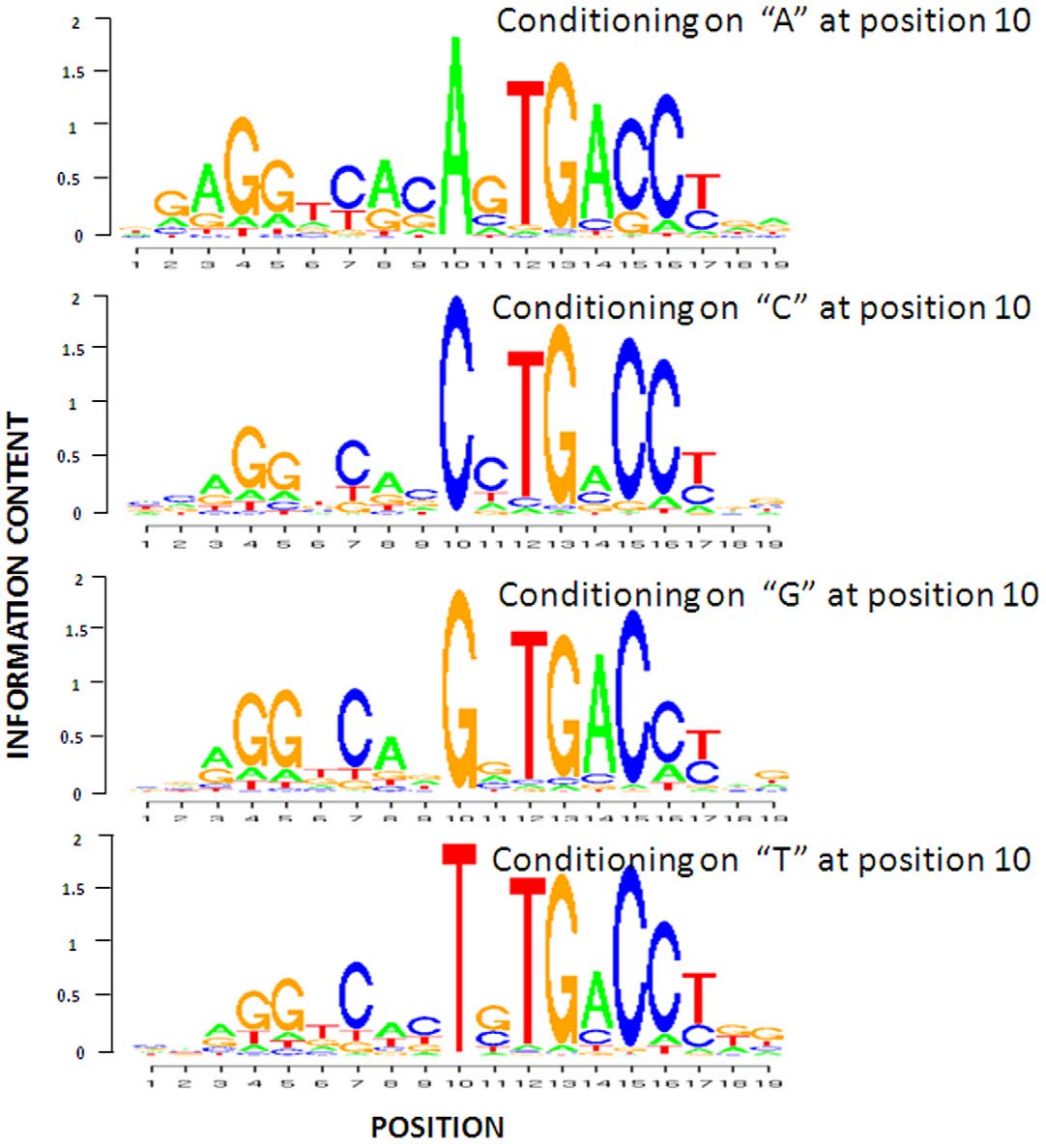
The conditional profiles of ER binding sites identified by TPD given that the nucleotide at position 10 is equal to A, C, G and T, respectively.

In Figure 8, each number in the rectangle next to some non-leaf nodes denotes the number of TFBSs available at that node. The first splitting position is 10 and totally 6291 sequences out of 10049 contain at least one TFBS for ER-alpha factor. That means the predicted true positive rate is 6291/10049 = 0.626 and the largest MCC value is achieved at that rate. The depth of the tree is 5 and the tree stops growing due to not enough number of sequences at each leaf node. The identified maximal dependent positions at different nodes could suggest the existence of the long range interactions and complicated dependent structure. To further investigate the motif patterns at different branches of this tree, we showed the logo plots of the motif models gained from the four subsets after the first splitting (split at position 10) in Figure 9.

The four logo plots have the similar consistent sequences except the splitting position 10, but the information content at several positions varies significantly, especially the adjacent positions , 9 and 11 and some distant position 6, 14 etc. The identified motif model of the largest “A” subset is more conserved compared to the other three models. The complicated dependent structure identified by TPD for ER-alpha could suggest that the overall motif is a mixture of several subclasses. Similar conclusion could be obtained for CTCF and NRSF (Figure S3, S4, S5 and S6).

Since the true motif is unknown, we used the enrichment of the predicted motif as a criterion to compare TPD with others. This is based on an assumption that among the multiple predicted motif patterns, the one that is most enriched in the ChIP-Seq candidate sequences relative to random control sequences is closest to the true motif pattern [32]. We used a cross-validation scheme to assess motif enrichment. The original positive dataset is equally divided into two halves: a training set and a testing set. The negative datasets are created by randomly shuffling the positive sets once. We ran TPD on positive and negative training datasets, using the PWM stored in TRANSFAC database as initial input. HMS, MEME and ChIPMunk were applied on the positive training dataset. We then switch the roles of these two halves and repeat the process.

**Figure 10:**
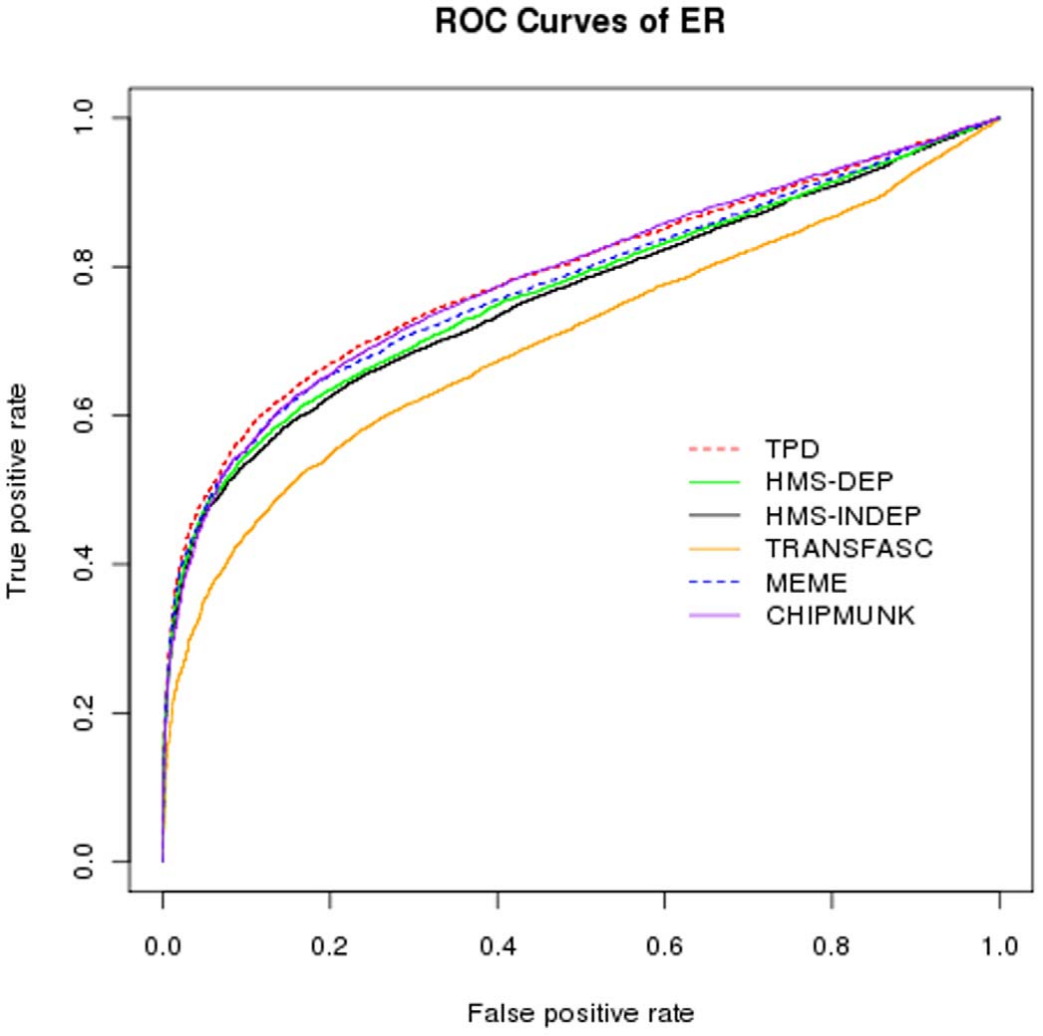
ROC curves of TPD, HMS-DEP, HMS, ChIPMunk, MEME and TRANSFAC for ER-alpha transcription factors.

The ROC curves for the ER-alpha factor based on those predicted motif patterns, as well as the ones stored in TRANSFAC database are shown in Figure 10. Two versions of HMS are applied here. HMS-INDEP assumes that all positions are independent and HMS-DEP allows up to triple intra-motif dependency. The ROC curves for NRSF and CTCF are shown in Figure S7 and S8. We then compute the AUCs and list them in Table 1. TPD has best performance in terms of sensitivity and specificity for both ER-alpha and CTCF transcription factors. With respect to NRSF, TPD is next to ChIPMunk.

**Table 1:** AUC values of TPD, HMS-DEP, HMS-INDEP, TRANSFAC, MEME and CHIPMUNK for different transcription factors.

	ER	CTCF	NRSF
TPD	0.788	0.941	0.795
HMS-DEP	0.766	0.934	0.788
HMS-INDEP	0.759	0.934	0.775
TRANSFAC	0.700	0.928	0.761
MEME	0.783	0.934	0.774
CHIPMUNK	0.774	0.935	0.809

For the ChIP-Seq data analysis, all computations are conducted on one node of a linux cluster (2.40 GHz CPU and 32GB RAM). We ran both MEME and ChIPMunk in parallel version with 4 threads. The computational times for all the methods are shown in Table 2. Note that the computational time for TPD does not change much as the number of input sequences increases. We ran TPD in a single core at this time and it could be easily implemented to support multiprocessor execution. It is also worthy of note that MEME with –con option is a potential good candidate for ChIP-Seq data analysis if there are no correlated positions in a motif.

**Table 2:** Computational time of TPD, HMS-DEP, MEME and CHIPMUNK for different transcription factors (in hours).

	ER	CTCF	NRSF
TPD	20	23	11
HMS-DEP	11.5	30	5.5
MEME	9.5	>2 days	1.5
CHIPMUNK	8.5	38	10

## Discussion

Due to the 3-D structure of the TFBS-proteins binding complex, some non-adjacent nucleotide positions could interact together to assemble the binding complex. This implies the possible existence of long range dependency in the motif pattern. Previous studies also have shown that the motif pattern of a single TF could be a mixture of multiple subtypes [5], [45]. The existence of such multiple subclasses could be one of the mechanisms which induce the complicated dependent structure of a single motif model. With the increasing volume of ChIP-Seq data available, it is possible now to investigate potential dependent structure existing in some motif models.

In this paper, we proposed a novel approach, known as TPWM, to model the interaction between DNA and transcription factor. We also modified an existing discriminative approach to construct the TPWM utilizing the corresponding PWM stored in TRANSFAC database as initial input and ChIP-Seq data. The simulation study showed that TPD can reliably and accurately predict the motif pattern. The output TPWM of TPD truly reflects the dependent structure of the simulated motif. Further comparison on real ChIP-Seq studies show that the identified motif patterns of TPD are more enriched or competitive in the ChIP-Seq data compared to recently developed ChIP-Seq data analysis tools, HMS [32] and ChIPMunk [32]. The proposed method to construct the TPWM from ChIP-Seq data requires initial input of the corresponding PWM. Usually those PWMs can be obtained from either TRANSFAC or JASPAR database [36]. If the PWM is unknown for a TF, de novo motif discovery algorithms, such as MEME, can be applied first to the top candidate peaks from ChIP-Seq data and then the predicted PWM can be used as initial input, and then feed all the ChIP-seq peaks to TPD to construct the corresponding TPWM structure.

One interesting extension of our method would be to incorporate variable length into the motif model. For example, it is well known that p-53 family members bind to DNA sequences with a variable spacer. In summary, we proposed a novel tree-based PWM approach to model the dependent structure in the TFBSs and successfully applied it on ChIP-seq datasets.

### Availability and Implementation

An initial Perl implement of our algorithm can be downloaded from http://bioinformatics.wistar.upenn.edu/TPD

## Supporting Information

Figure S1 **Four independent motif models for two motif widths and two degree of conservation used in the simulation study.**
(TIF)

Figure S2 **TPWMs of the predicted simulated dependent motif model by TPD for the strong, abundant and 6 correlated positions (3,4,12,13,19,20) model for each of the 10 test datasets (6000 sequences for each data set).**
(TIF)

Figure S3 **Predicted TPWM by TPD for NRSF.**
(TIF)

Figure S4 **Predicted TPWM by TPD for CTCF.**
(TIF)

Figure S5 **The conditional profiles of NRSF binding sites identified by TPD given that the nucleotide at position 11 is equal to A, C, G and T, respectively.**
(TIF)

Figure S6 **The conditional profiles of CTCF binding sites identified by TPD given that the nucleotide at position 17 is equal to A, C, G and T, respectively.**
(TIF)

Figure S7 **ROC curves of TPD, HMS-DEP, HMS and TRANSFAC for NRSF factor.**
(TIF)

Figure S8 **ROC curves of TPD, HMS-DEP, HMS and TRANSFAC for CTCF factor.**
(TIF)

Table S1 **Four independent motif models for two motif width and two motif strengths used in the simulation study.**
(DOC)

Table S2 **Two motif abundances scheme used in simulation table.**
(DOC)

Table S3 **Six dependent motif models for two motif width and two motif strengths used in the simulation study.**
(DOC)

Table S4 **The probability distributions for the dependent motif patterns with width equal to 10.**
(DOC)

Table S5 **The probability distributions for the dependent motif patterns with width equal to 20 and 4 correlated positions.**
(DOC)

Table S6 **The probability distributions for the dependent motif patterns with width equal to 20 and 6 correlated positions.**
(DOC)

Table S7 **Predicted true positive rates by TPD for the simulation study with independent motif models.**
(DOC)

Table S8 **Predicted true positive rates by TPD for the simulation study with dependent motif models.**
(DOC)
