# Supplementary figures and images for: Tree-Based Position Weight Matrix Approach to Model Transcription Factor Binding Site Profiles
Source: PLoS One. 2011 Sep 2;6(9):e24210. doi: 10.1371/journal.pone.0024210 (PMC3166302; doi:10.1371/journal.pone.0024210)

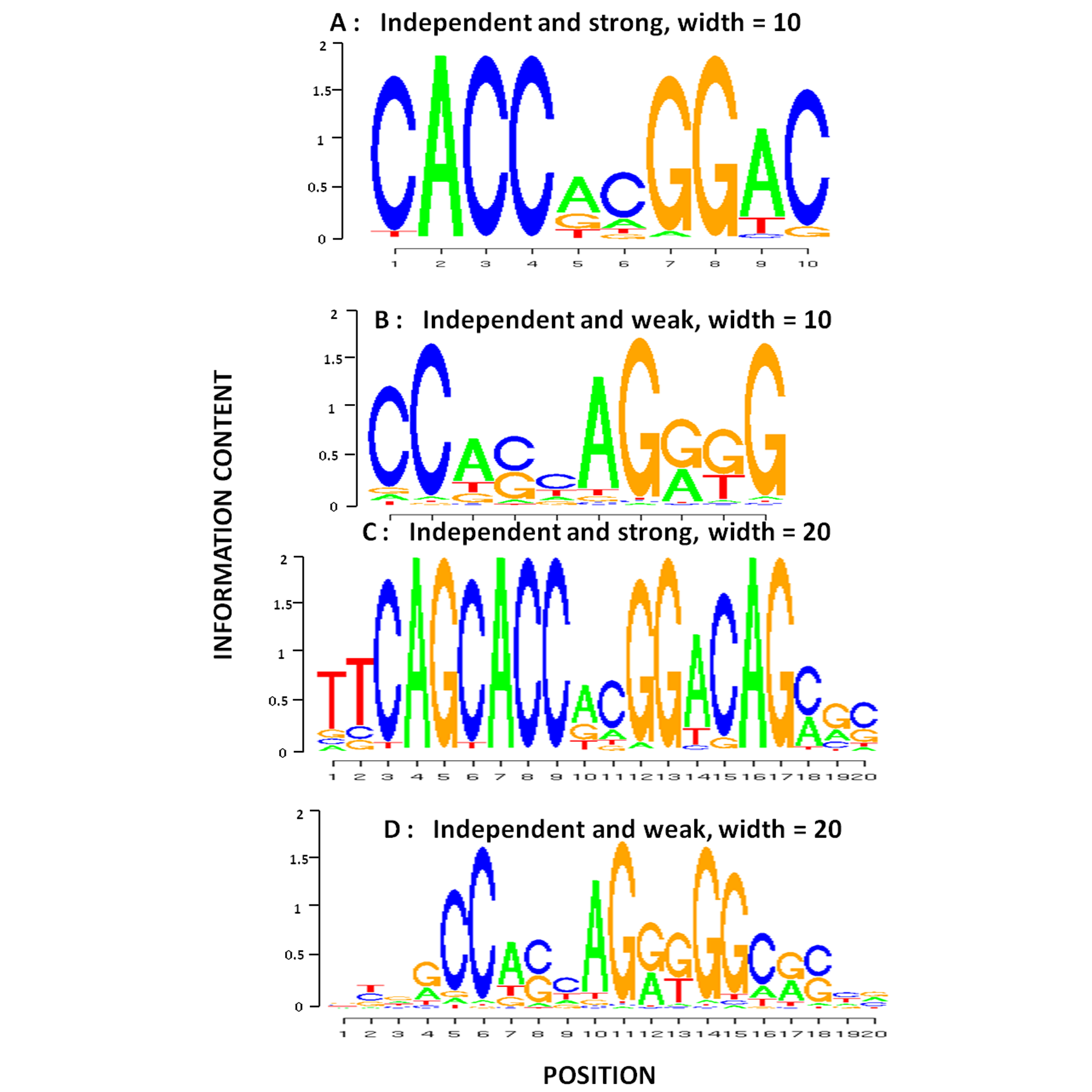

Supplement: Figure S1 — Four independent motif models for two motif widths and two degree of conservation used in the simulation study. (TIF) [file pone.0024210.s001.tif]

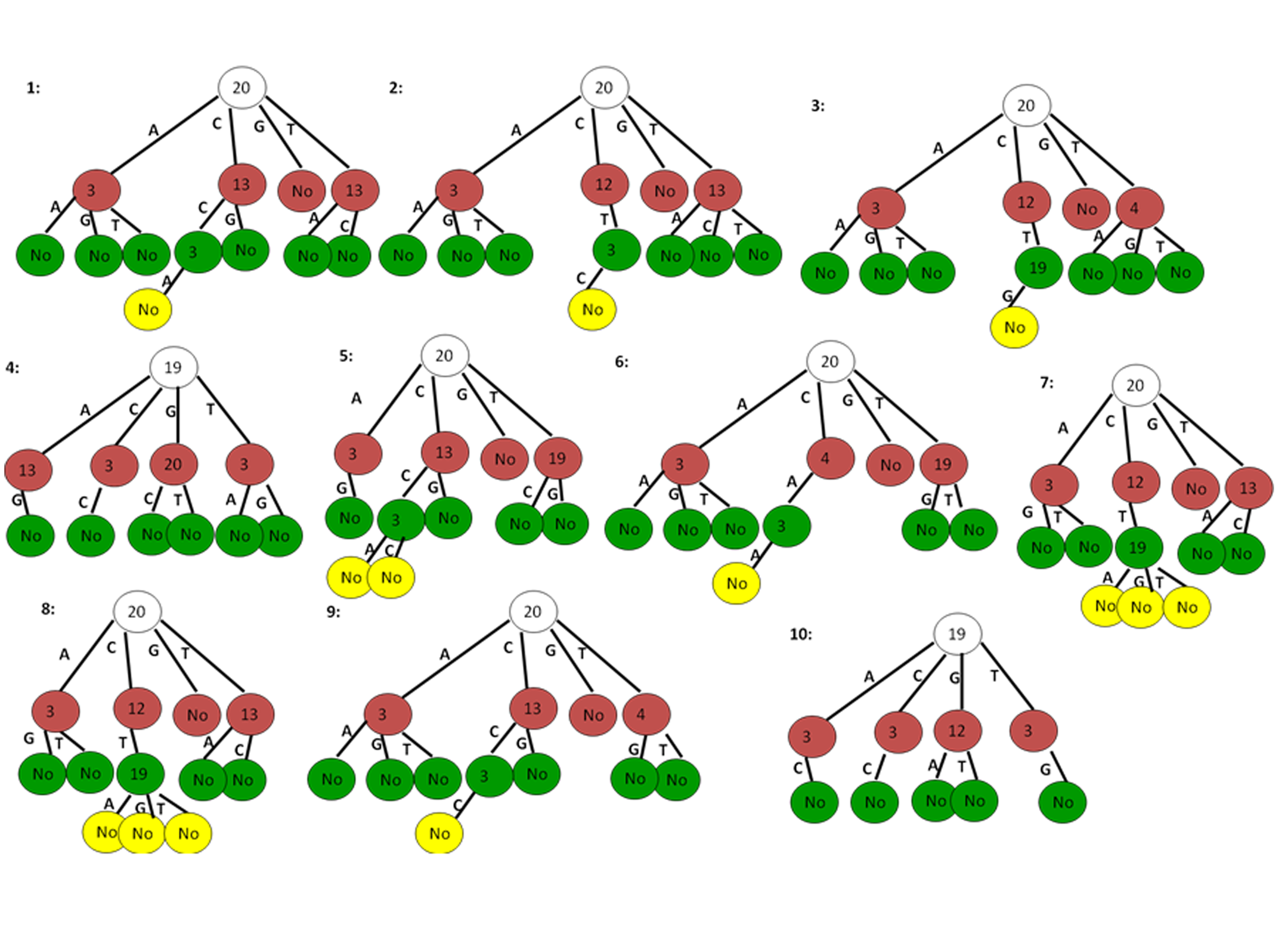

Supplement: Figure S2 — TPWMs of the predicted simulated dependent motif model by TPD for the strong, abundant and 6 correlated positions (3,4,12,13,19,20) model for each of the 10 test datasets (6000 sequences for each data set). (TIF) [file pone.0024210.s002.tif]

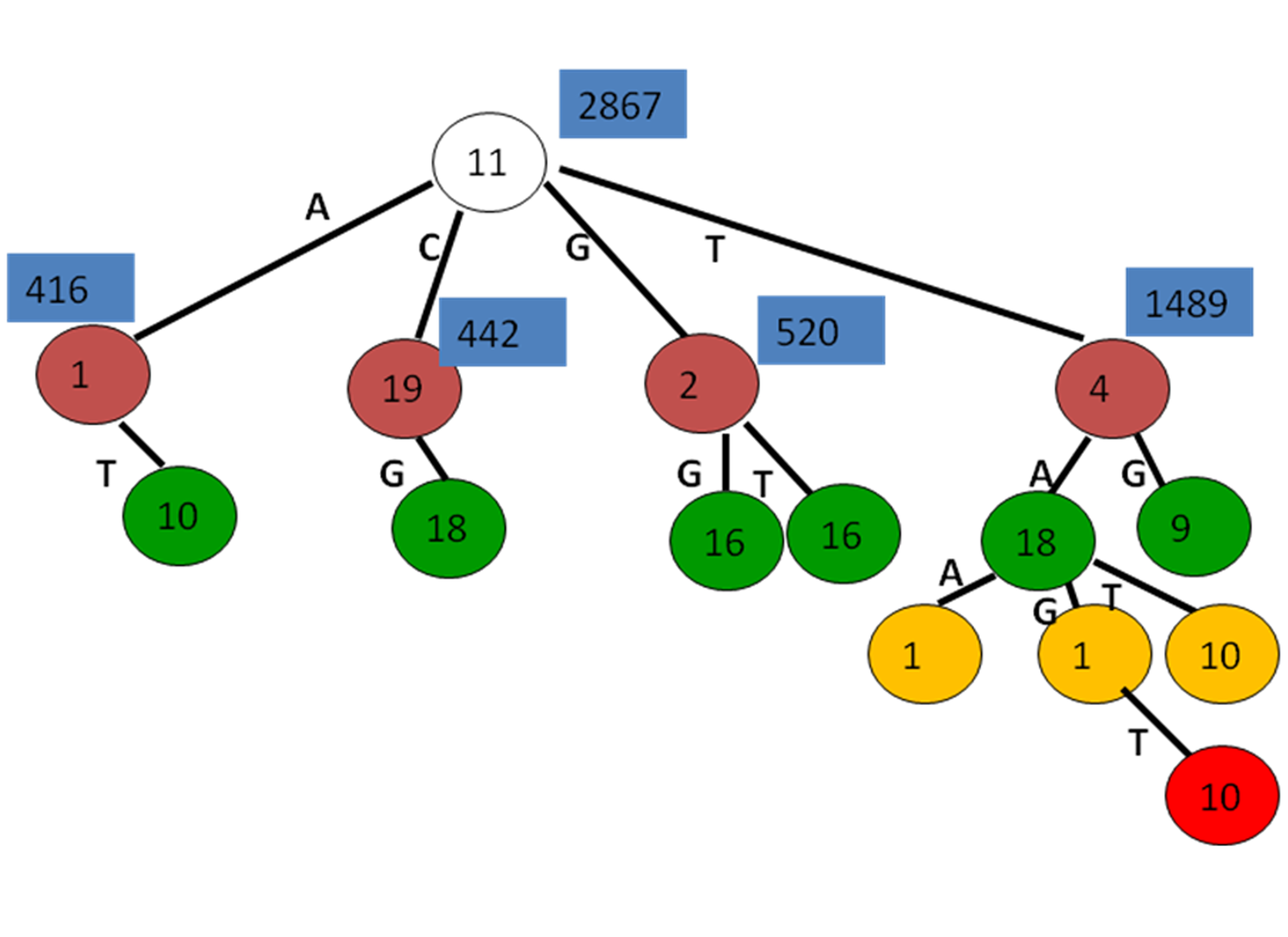

Supplement: Figure S3 — Predicted TPWM by TPD for NRSF. (TIF) [file pone.0024210.s003.tif]

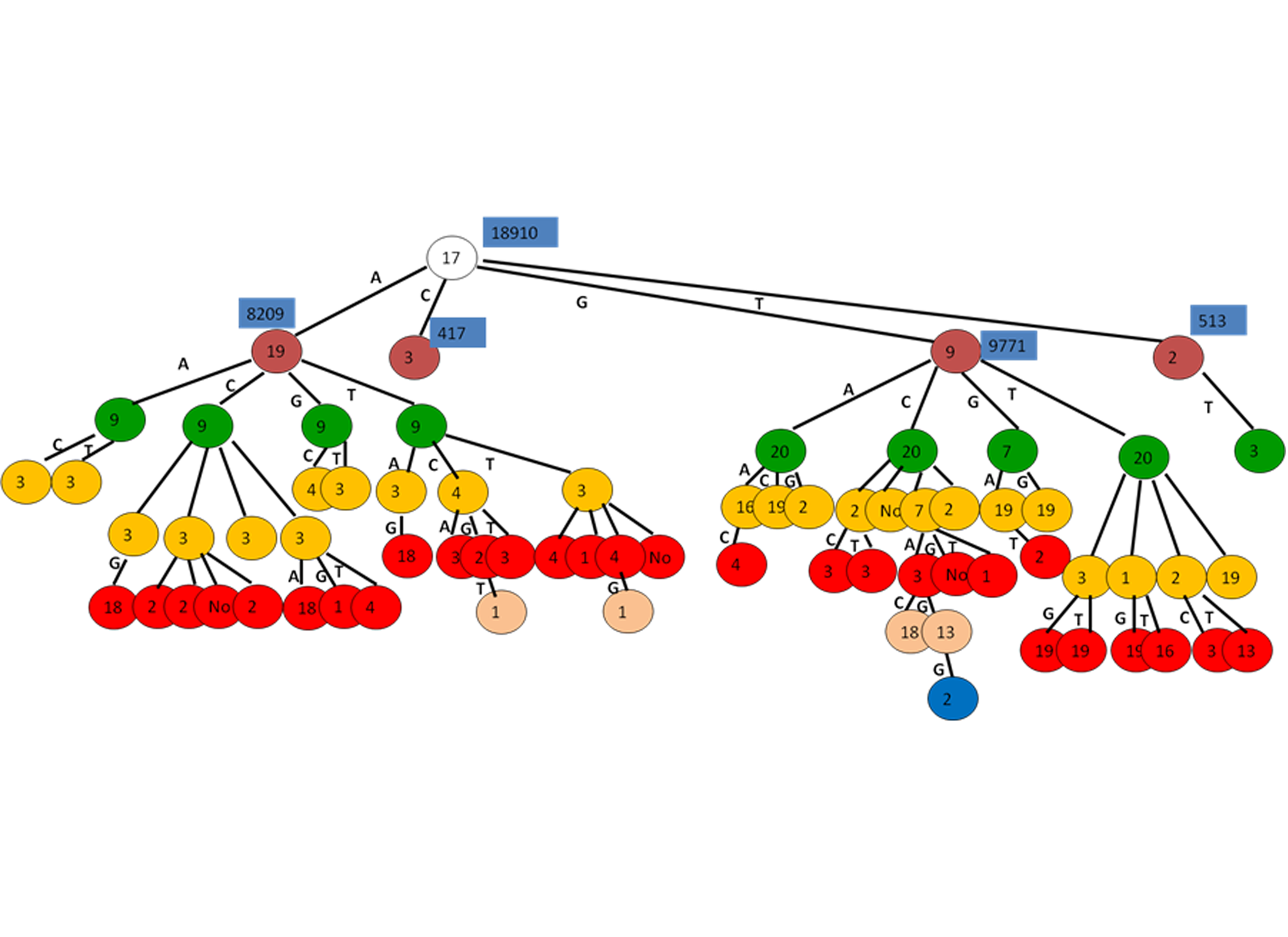

Supplement: Figure S4 — Predicted TPWM by TPD for CTCF. (TIF) [file pone.0024210.s004.tif]

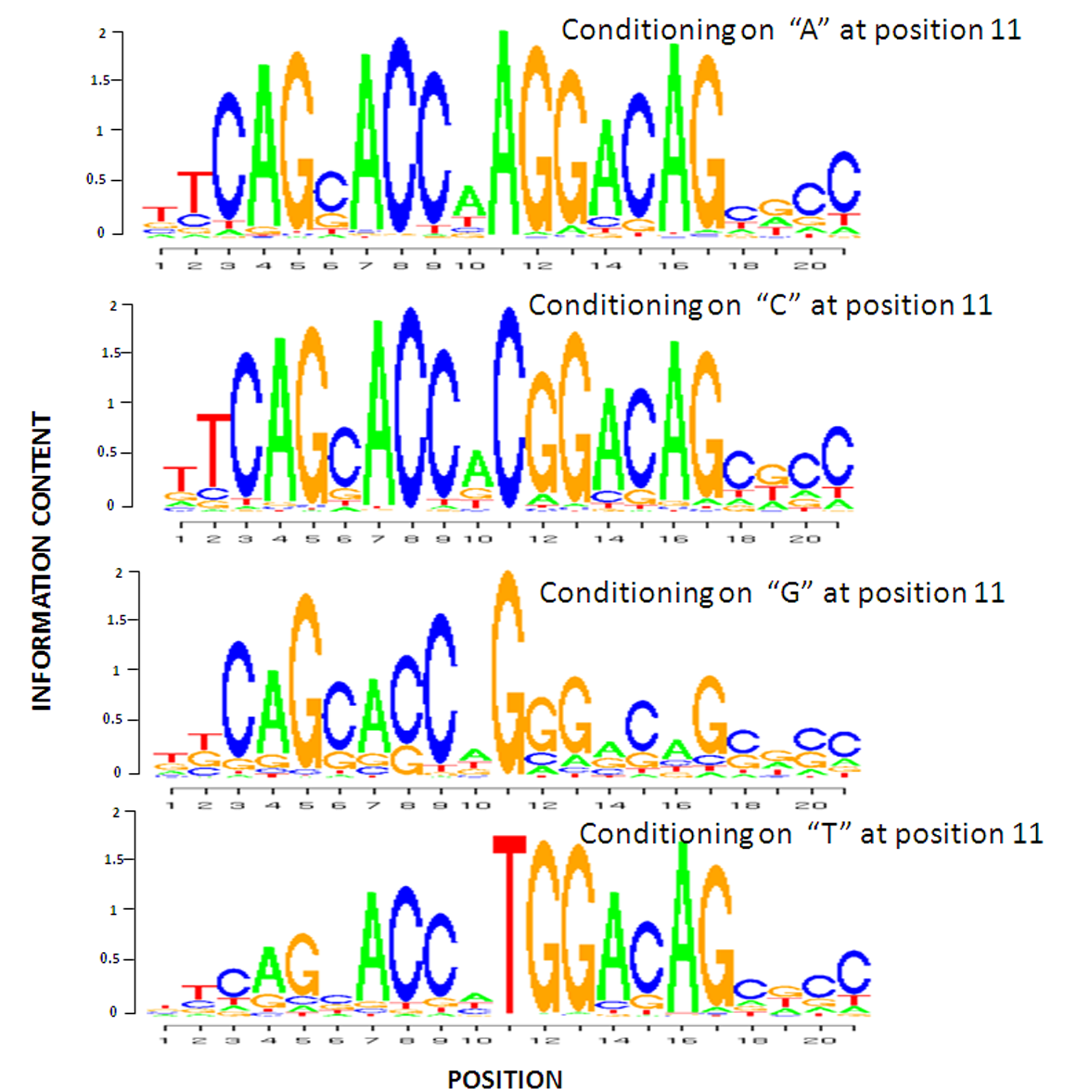

Supplement: Figure S5 — The conditional profiles of NRSF binding sites identified by TPD given that the nucleotide at position 11 is equal to A, C, G and T, respectively. (TIF) [file pone.0024210.s005.tif]

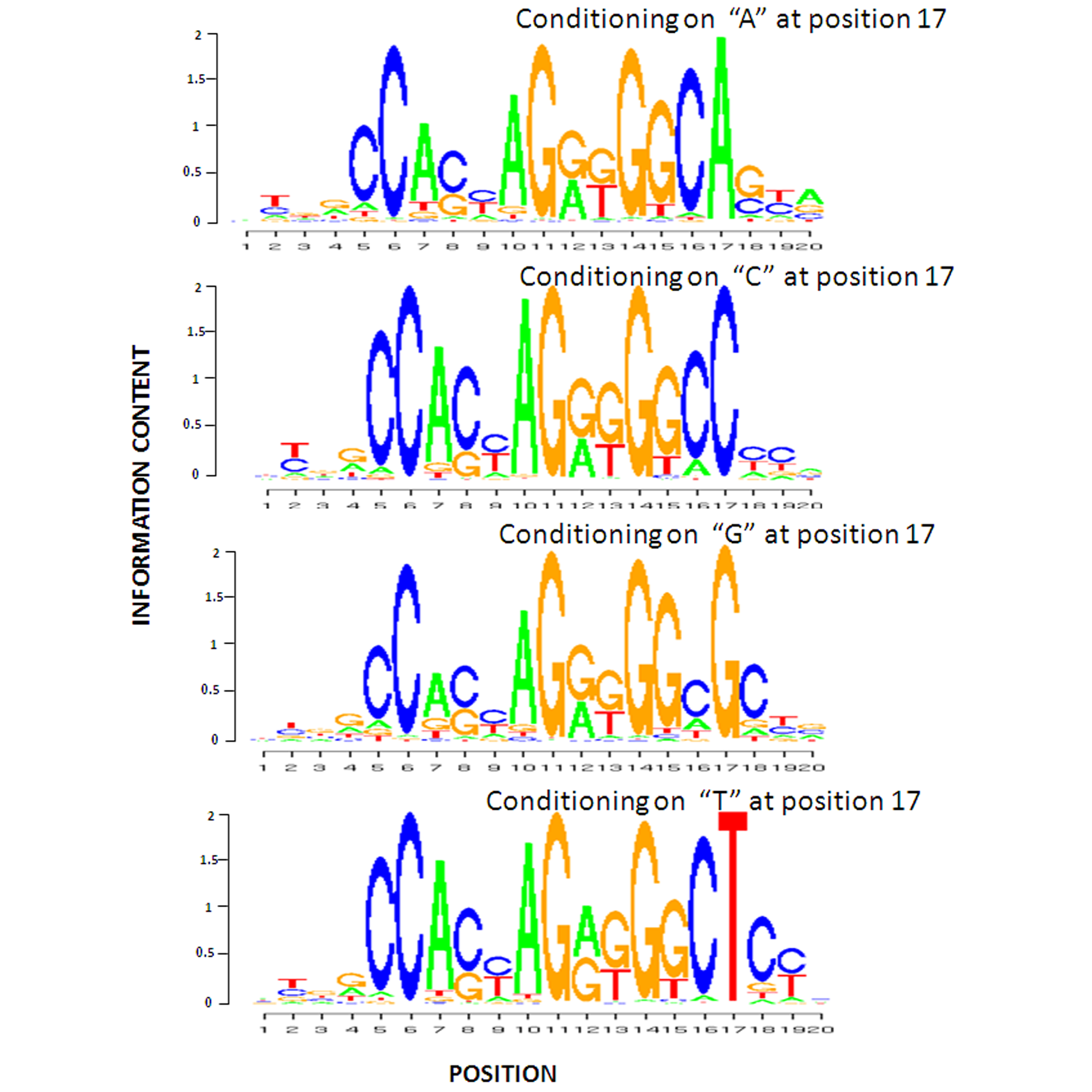

Supplement: Figure S6 — The conditional profiles of CTCF binding sites identified by TPD given that the nucleotide at position 17 is equal to A, C, G and T, respectively. (TIF) [file pone.0024210.s006.tif]

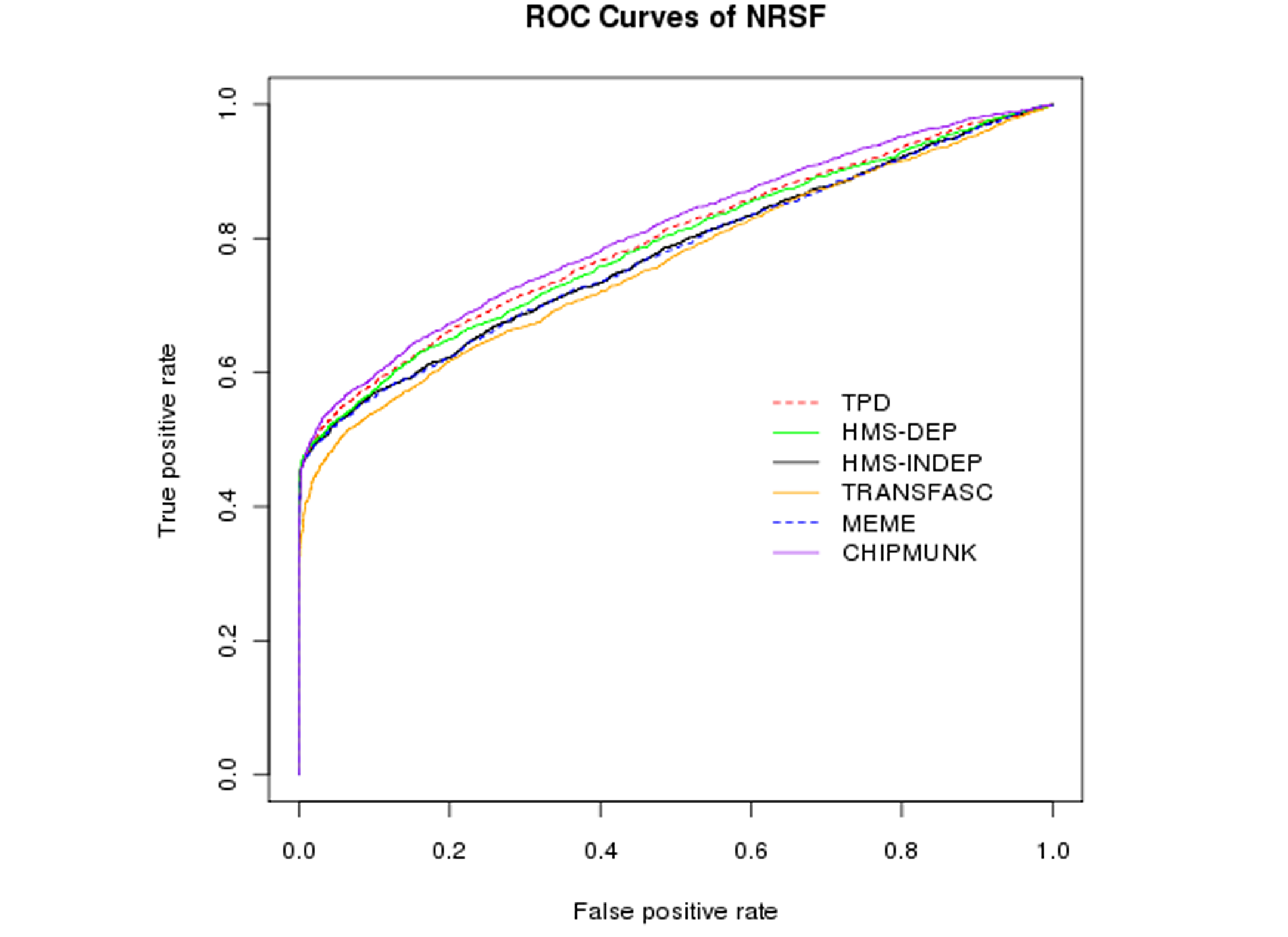

Supplement: Figure S7 — ROC curves of TPD, HMS-DEP, HMS and TRANSFAC for NRSF factor. (TIF) [file pone.0024210.s007.tif]

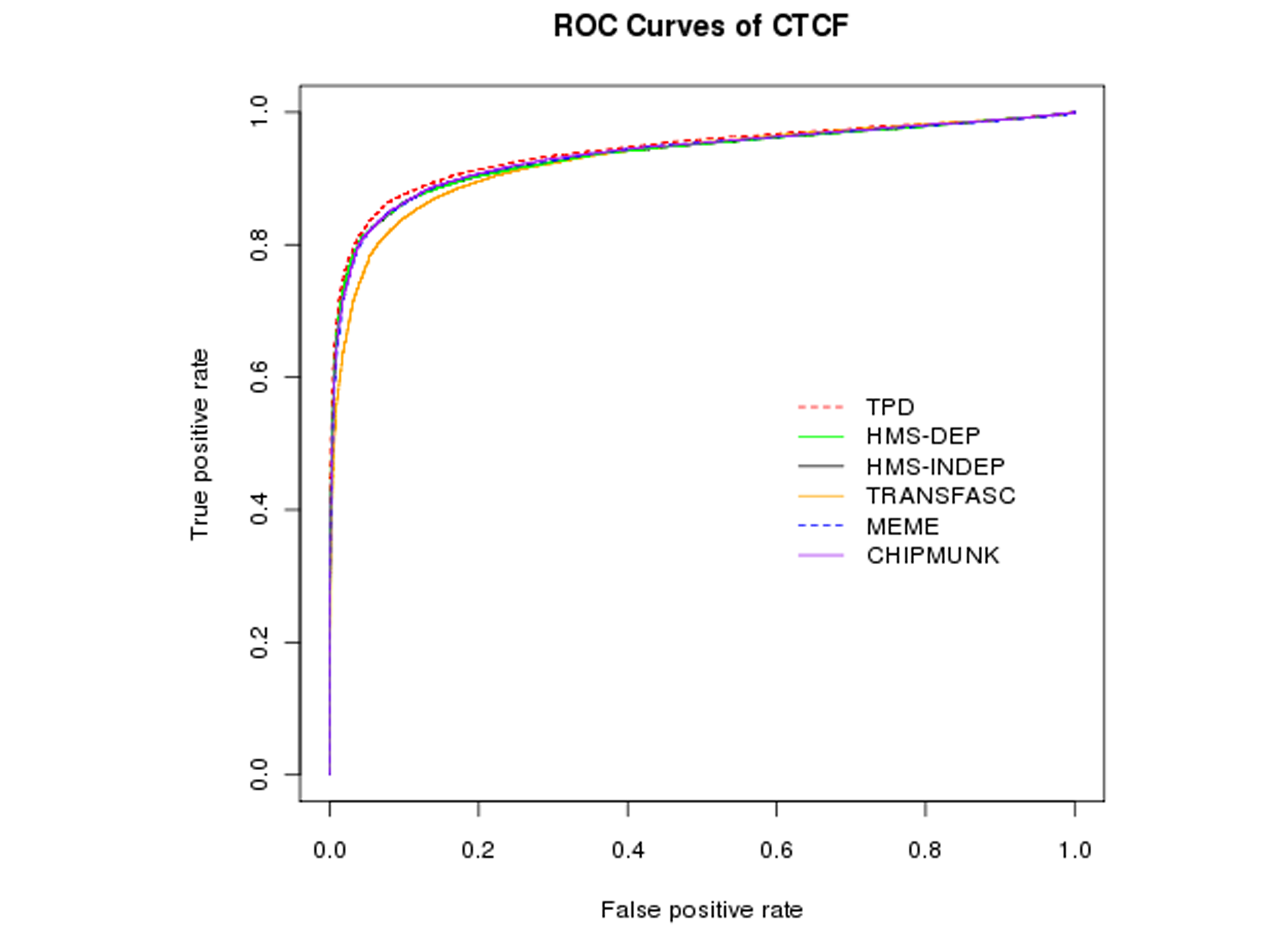

Supplement: Figure S8 — ROC curves of TPD, HMS-DEP, HMS and TRANSFAC for CTCF factor. (TIF) [file pone.0024210.s008.tif]
