# Supplementary material for: Tree-Based Position Weight Matrix Approach to Model Transcription Factor Binding Site Profiles
Source: PLoS One. 2011 Sep 2;6(9):e24210. doi: 10.1371/journal.pone.0024210 (PMC3166302; doi:10.1371/journal.pone.0024210)
Supplement: Table S1 — Four independent motif models for two motif width and two motif strengths used in the simulation study. (DOC) [file pone.0024210.s009.doc]

**Table S1. Four independent motif models for two motif width and two motif strengths used in the simulation study.**

| Motif width | Motif information content | |
| --- | --- | --- |
|  | Strong | Weak |
| 10 | 1.57 | 1.11 |
| 20 | 1.45 | 0.78 |
