# Supplementary material for: Tree-Based Position Weight Matrix Approach to Model Transcription Factor Binding Site Profiles
Source: PLoS One. 2011 Sep 2;6(9):e24210. doi: 10.1371/journal.pone.0024210 (PMC3166302; doi:10.1371/journal.pone.0024210)
Supplement: Table S2 — Two motif abundances scheme used in simulation table. (DOC) [file pone.0024210.s010.doc]

**Table S2. Two motif abundances scheme used in simulation table.**

| Total # of sequences | Sequence length | Motif abundance |
| --- | --- | --- |
| 3000 | 200 | 80% |
| 3000 | 200 | 50% |
