# Supplementary material for: Tree-Based Position Weight Matrix Approach to Model Transcription Factor Binding Site Profiles
Source: PLoS One. 2011 Sep 2;6(9):e24210. doi: 10.1371/journal.pone.0024210 (PMC3166302; doi:10.1371/journal.pone.0024210)
Supplement: Table S3 — Six dependent motif models for two motif width and two motif strengths used in the simulation study. (DOC) [file pone.0024210.s011.doc]

**Table S3**. Six dependent motif models for two motif width and two motif strengths used in the simulation study.

| Motif width | Motif information content | Correlated Positions |
| --- | --- | --- |
| 20 | strong | 3,4,12,13,19,20 |
|  |  | 12,13,19,20 |
| 20 | weak | 2,3,4,9,19,20 |
|  |  | 3,4,18,19 |
| 10 | strong | 5,9 |
| 10 | weak | 3,9 |
