# Supplementary material for: Tree-Based Position Weight Matrix Approach to Model Transcription Factor Binding Site Profiles
Source: PLoS One. 2011 Sep 2;6(9):e24210. doi: 10.1371/journal.pone.0024210 (PMC3166302; doi:10.1371/journal.pone.0024210)
Supplement: Table S4 — The probability distributions for the dependent motif patterns with width equal to 10. (DOC) [file pone.0024210.s012.doc]

**Table S4.** The probability distributions for the dependent motif patterns with width equal to 10.

| Width = 10 (2 correlated positions) | | | |
| --- | --- | --- | --- |
| Strong (5 and 9) | | Weak(3 and 9) | |
| Nucleotides combination | probability | Nucleotides combination | probability |
| GG | 0.5 | AT | 0.5 |
| TC | 0.5 | GA | 0.5 |
