# Supplementary material for: Tree-Based Position Weight Matrix Approach to Model Transcription Factor Binding Site Profiles
Source: PLoS One. 2011 Sep 2;6(9):e24210. doi: 10.1371/journal.pone.0024210 (PMC3166302; doi:10.1371/journal.pone.0024210)
Supplement: Table S5 — The probability distributions for the dependent motif patterns with width equal to 20 and 4 correlated positions. (DOC) [file pone.0024210.s013.doc]

**Table S5.** The probability distributions for the dependent motif patterns with width equal to 20 and 4 correlated positions.

| Width = 20 (4 correlated positions) | | | |
| --- | --- | --- | --- |
| Strong(12,13,19,20) | | Weak(3,4,18,19) | |
| Nucleotides combination | probability | Nucleotides combination | probability |
| CTTT | 1/3 | GCCT | 1/3 |
| TAAT | 1/3 | ACGT | 1/3 |
| AAAA | 1/12 | GCTT | 1/12 |
| CATA | 1/12 | TCGC | 1/12 |
| ATTT | 1/12 | CTCG | 1/12 |
| AGAC | 1/12 | GCCA | 1/12 |
