# Supplementary material for: Tree-Based Position Weight Matrix Approach to Model Transcription Factor Binding Site Profiles
Source: PLoS One. 2011 Sep 2;6(9):e24210. doi: 10.1371/journal.pone.0024210 (PMC3166302; doi:10.1371/journal.pone.0024210)
Supplement: Table S6 — The probability distributions for the dependent motif patterns with width equal to 20 and 6 correlated positions. (DOC) [file pone.0024210.s014.doc]

**Table S6.** The probability distributions for the dependent motif patterns with width equal to 20 and 6 correlated positions.

| Width = 20 (6 correlated positions) | | | |
| --- | --- | --- | --- |
| Strong(3,4,12,13,19,20) | | Weak(2,3,4,9,19,20) | |
| Nucleotides combination | Probability | Nucleotides combination | probability |
| CTTTTT | 1/24 | GTCTAC | 1/24 |
| TACACA | 1/24 | GACGGG | 1/24 |
| AAAATA | 1/24 | TGCGCA | 1/24 |
| CATCAC | 1/24 | GTAGAC | 1/24 |
| CAAACT | 1/24 | ATGATA | 1/24 |
| AGAGAC | 1/24 | AGACAC | 1/24 |
| AATCGC | 1/24 | CTTGTG | 1/24 |
| TTGTTG | 1/24 | ACAAAT | 1/24 |
| GGACGT | 1/3 | ATCGCC | 1/3 |
| GACCTA | 1/3 | CAGAGT | 1/3 |
