# Supplementary material for: Tree-Based Position Weight Matrix Approach to Model Transcription Factor Binding Site Profiles
Source: PLoS One. 2011 Sep 2;6(9):e24210. doi: 10.1371/journal.pone.0024210 (PMC3166302; doi:10.1371/journal.pone.0024210)
Supplement: Table S7 — Predicted true positive rates by TPD for the simulation study with independent motif models. (DOC) [file pone.0024210.s015.doc]

**Table S7.** Predicted true positive rates by TPD for the simulation study with independent motif models.

| Width = 10 | | | |  | Width = 20 | | | |
| --- | --- | --- | --- | --- | --- | --- | --- | --- |
| strong | | Weak | |  | strong | | weak | |
| abundant | sparse | abundant | sparse |  | abundant | sparse | abundant | sparse |
| 0.803 | 0.493 | 0.616 | 0.407 |  | 0.804 | 0.510 | 0.722 | 0.462 |
| 0.770 | 0.510 | 0.616 | 0.372 |  | 0.813 | 0.514 | 0.740 | 0.445 |
| 0.782 | 0.503 | 0.657 | 0.409 |  | 0.800 | 0.508 | 0.761 | 0.460 |
| 0.794 | 0.497 | 0.625 | 0.485 |  | 0.808 | 0.495 | 0.726 | 0.434 |
| 0.779 | 0.503 | 0.674 | 0.337 |  | 0.795 | 0.527 | 0.736 | 0.457 |
| 0.794 | 0.493 | 0.658 | 0.394 |  | 0.811 | 0.510 | 0.733 | 0.457 |
| 0.769 | 0.491 | 0.641 | 0.382 |  | 0.803 | 0.513 | 0.721 | 0.442 |
| 0.786 | 0.524 | 0.589 | 0.361 |  | 0.817 | 0.502 | 0.737 | 0.481 |
| 0.782 | 0.495 | 0.576 | 0.339 |  | 0.803 | 0.497 | 0.755 | 0.460 |
| 0.774 | 0.499 | 0.633 | 0.379 |  | 0.812 | 0.500 | 0.742 | 0.455 |
