# Supplementary material for: Tree-Based Position Weight Matrix Approach to Model Transcription Factor Binding Site Profiles
Source: PLoS One. 2011 Sep 2;6(9):e24210. doi: 10.1371/journal.pone.0024210 (PMC3166302; doi:10.1371/journal.pone.0024210)
Supplement: Table S8 — Predicted true positive rates by TPD for the simulation study with dependent motif models. (DOC) [file pone.0024210.s016.doc]

**Table S8.** Predicted true positive rates by TPD for the simulation study with dependent motif models.

| Width = 20 (6 correlated positions) | | | |  | Width = 20(4 correlated positions) | | | |
| --- | --- | --- | --- | --- | --- | --- | --- | --- |
| strong | | weak | |  | strong | | weak | |
| abundant | sparse | abundant | sparse |  | abundant | sparse | abundant | sparse |
| 0.796 | 0.49 | 0.815 | 0.523 |  | 0.803 | 0.501 | 0.776 | 0.494 |
| 0.790 | 0.511 | 0.799 | 0.520 |  | 0.806 | 0.508 | 0.798 | 0.485 |
| 0.800 | 0.482 | 0.784 | 0.541 |  | 0.806 | 0.500 | 0.788 | 0.492 |
| 0.789 | 0.522 | 0.794 | 0.541 |  | 0.800 | 0.488 | 0.767 | 0.498 |
| 0.790 | 0.515 | 0.783 | 0.518 |  | 0.792 | 0.502 | 0.775 | 0.499 |
| 0.805 | 0.522 | 0.807 | 0.528 |  | 0.795 | 0.516 | 0.772 | 0.497 |
| 0.811 | 0.499 | 0.793 | 0.506 |  | 0.797 | 0.503 | 0.797 | 0.510 |
| 0.792 | 0.506 | 0.794 | 0.506 |  | 0.800 | 0.501 | 0.764 | 0.500 |
| 0.801 | 0.512 | 0.781 | 0.539 |  | 0.807 | 0.524 | 0.762 | 0.482 |
| 0.811 | 0.507 | 0.783 | 0.502 |  | 0.796 | 0.513 | 0.781 | 0.483 |

| Width = 10 (2 correlated positions) | | | |
| --- | --- | --- | --- |
| strong | | weak | |
| abundant | sparse | abundant | sparse |
| 0.818 | 0.522 | 0.748 | 0.499 |
| 0.807 | 0.530 | 0.681 | 0.499 |
| 0.815 | 0.516 | 0.717 | 0.477 |
| 0.805 | 0.525 | 0.732 | 0.498 |
| 0.805 | 0.523 | 0.723 | 0.534 |
| 0.808 | 0.532 | 0.698 | 0.49 |
| 0.816 | 0.604 | 0.705 | 0.516 |
| 0.797 | 0.536 | 0.687 | 0.494 |
| 0.806 | 0.512 | 0.712 | 0.466 |
| 0.813 | 0.534 | 0.719 | 0.488 |
